# Supplementary material for: Hybrid ARIMA-LSTM for COVID-19 forecasting: a comparative AI modeling study
Source: PeerJ Comput Sci. 2025 Sep 19;11:e3195. doi: 10.7717/peerj-cs.3195 (PMC12453849; doi:10.7717/peerj-cs.3195)
Supplement: Supplemental Information 2 [file peerj-cs-11-3195-s002.docx]

**Supplementary table 1:**

**ARIMA Parameter Selection and Model Performance of Active Cases**

| **Models** | **Metrics** | | | | | | |
| --- | --- | --- | --- | --- | --- | --- | --- |
|  | **MSE** | **RMSE** | **Relative RMSE (RRMSE)** | **Normalized RMSE (NRMSE)** | **MAE** | **MAPE (%)** | **R²** |
| ARIMA (0, 3, 7) | 4073554.02 | 2018.30 | 0.2260 | 0.0999 | 1587.09 | 7.68% | -0.1258 |
| ARIMA (2, 3, 8) | 3924280.95 | 1980.98 | 0.2218 | 0.0981 | 1542.03 | 7.58% | -0.0845 |
| ARIMA (3, 1, 6) | 3712254.96 | 1926.72 | 0.2158 | 0.0954 | 1608.16 | 7.83% | -0.0259 |
| ARIMA (5, 1, 5) | 3653469.62 | 1911.41 | 0.2140 | 0.0947 | 1564.49 | 7.86% | -0.0097 |
| ARIMA (6, 1, 6) | 3451941.18 | 1857.94 | 0.2081 | 0.0920 | 1537.18 | 7.55% | 0.0460 |
| …… | …… | …… | …… | …… | …… | …… | …… |
| **ARIMA (7, 4, 6)** | **2568836.02** | **1602.76** | **0.1795** | **0.0794** | **1325.89** | **6.55%** | **0.2901** |
| **LSTM**  **(Epochs=200,**  **Batch size=16,**  **Verbose=1)** | **3352686.45** | **1831.03** | **0.2050** | **0.0919** | **1545.04** | **8.09%** | **0.2044** |
| **Hybrid ARIMA-LSTM (p= 7, d=4, q=6 & Epochs=200,**  **Batch size=16,**  **Verbose=1)** | **2501541.09** | **1581.63** | **0.1771** | **0.0783** | **1297.84** | **6.43%** | **0.3087** |
