# Supplementary material for: Hybrid ARIMA-LSTM for COVID-19 forecasting: a comparative AI modeling study
Source: PeerJ Comput Sci. 2025 Sep 19;11:e3195. doi: 10.7717/peerj-cs.3195 (PMC12453849; doi:10.7717/peerj-cs.3195)
Supplement: Supplemental Information 3 [file peerj-cs-11-3195-s003.docx]

**Supplementary table 2:**

**ARIMA Parameter Selection and Model Performance of Recovery Cases**

| **Models** | **Metrics** | | | | | | |
| --- | --- | --- | --- | --- | --- | --- | --- |
|  | **MSE** | **RMSE** | **Relative RMSE (RRMSE)** | **Normalized RMSE (NRMSE)** | **MAE** | **MAPE (%)** | **R²** |
| ARIMA (0, 3, 4) | 6075398.65 | 2464.83 | 0.2939 | 0.1228 | 1815.33 | 9.17% | -0.2088 |
| ARIMA (1, 1, 4) | 4826535.35 | 2196.94 | 0.2619 | 0.1095 | 1632.87 | 8.45% | 0.0397 |
| ARIMA (1, 1, 5) | 4627841.69 | 2151.24 | 0.2565 | 0.1072 | 1607.78 | 8.29% | 0.0792 |
| ARIMA (1, 1, 6) | 4127170.67 | 2031.54 | 0.2422 | 0.1012 | 1623.19 | 7.95% | 0.1788 |
| ARIMA (3, 1, 8) | 4043638.38 | 2010.88 | 0.2397 | 0.1002 | 1562.50 | 7.86% | 0.1954 |
| …… | …… | …… | …… | …… | …… | …… | …… |
| **ARIMA (8, 1, 2)** | **3974437.15** | **1993.60** | **0.2377** | **0.0993** | **1584.16** | **8.07%** | **0.2092** |
| **LSTM**  **(Epochs=200,**  **Batch size=16,**  **Verbose=1)** | **8280420.05** | **2877.57** | **0.3635** | **0.1394** | **2239.65** | **11.35%** | **- 0.8905** |
| **Hybrid ARIMA-LSTM (p= 8, d=1, q=2 & Epochs=200,**  **Batch size=16,**  **Verbose=1)** | **3973296.51** | **1993.31** | **0.2376** | **0.0993** | **1583.28** | **8.06%** | **0.2100** |
