# Supplementary material for: Hybrid ARIMA-LSTM for COVID-19 forecasting: a comparative AI modeling study
Source: PeerJ Comput Sci. 2025 Sep 19;11:e3195. doi: 10.7717/peerj-cs.3195 (PMC12453849; doi:10.7717/peerj-cs.3195)
Supplement: Supplemental Information 4 [file peerj-cs-11-3195-s004.docx]

**Supplementary table 3:**

**ARIMA Parameter Selection and Model Performance of Death Cases**

| **Models** | **Metrics** | | | | | | |
| --- | --- | --- | --- | --- | --- | --- | --- |
|  | **MSE** | **RMSE** | **Relative RMSE (RRMSE)** | **Normalized RMSE (NRMSE)** | **MAE** | **MAPE (%)** | **R²** |
| ARIMA (0, 1, 3) | 8612.77 | 92.81 | 0.2220 | 0.3072 | 66.21 | 19.75% | -0.2917 |
| ARIMA (0, 1, 4) | 8006.01 | 89.48 | 0.2141 | 0.2962 | 63.20 | 19.08% | -0.2007 |
| ARIMA (0, 1, 5) | 7760.21 | 88.09 | 0.2107 | 0.2916 | 62.11 | 18.92% | -0.1638 |
| ARIMA (0, 2, 4) | 7544.57 | 86.86 | 0.2078 | 0.2875 | 74.32 | 27.30% | -0.1315 |
| ARIMA (0, 2, 5) | 6849.24 | 82.76 | 0.1980 | 0.2739 | 70.21 | 25.95% | -0.0272 |
| …… | …… | …… | …… | …… | …… | …… | …… |
| **ARIMA (0, 3, 2)** | **3606.44** | **60.05** | **0.1437** | **0.1988** | **43.60** | **14.39%** | **0.4591** |
| **LSTM**  **(Epochs=200,**  **Batch size=16,**  **Verbose=1)** | **24307.72** | **155.91** | **0.3730** | **0.4881** | **124.89** | **42.06%** | **-2.3964** |
| **Hybrid ARIMA-LSTM (p= 0, d=3, q=2 & Epochs=200,**  **Batch size=16,**  **Verbose=1)** | **3524.56** | **59.37** | **0.1420** | **0.1965** | **45.08** | **15.28%** | **0.4714** |
