# Supplementary material for: Hybrid ARIMA-LSTM for COVID-19 forecasting: a comparative AI modeling study
Source: PeerJ Comput Sci. 2025 Sep 19;11:e3195. doi: 10.7717/peerj-cs.3195 (PMC12453849; doi:10.7717/peerj-cs.3195)
Supplement: Supplemental Information 5 [file peerj-cs-11-3195-s005.docx]

**Supplementary table 4:**

**LSTM Model parameter for Active, Recovery, and Death cases**

| **Parameter** | **Value** |
| --- | --- |
| LSTM Units | 200 |
| Batch Units | 200 |
| Epochs | 16 |
| Verbose | 1 |
| Optimization Approach | Adam |
