# Supplementary material for: Hybrid ARIMA-LSTM for COVID-19 forecasting: a comparative AI modeling study
Source: PeerJ Comput Sci. 2025 Sep 19;11:e3195. doi: 10.7717/peerj-cs.3195 (PMC12453849; doi:10.7717/peerj-cs.3195)
Supplement: Supplemental Information 6 [file peerj-cs-11-3195-s006.docx]

**Supplementary table 5:**

**Diebold-Mariano (DM) Test Results Comparing Forecast Accuracy Between Models**

| **Forecast Type** | **Model Comparison** | **DM Statistic** | **p-value** |
| --- | --- | --- | --- |
| Active Cases | ARIMA vs. LSTM | 1.5015 | 0.1839 |
|  | ARIMA vs. Hybrid | 1.3849 | 0.2154 |
|  | LSTM vs. Hybrid | 0.9631 | 0.3727 |
| Death Cases | ARIMA vs. LSTM | -0.8501 | 0.4279 |
|  | ARIMA vs. Hybrid | 3.1088 | **0.0209** |
|  | LSTM vs. Hybrid | -1.6500 | 0.2 |
| Recovery Cases | Hybrid vs. LSTM | -0.9631 | 0.3727 |
|  | Hybrid vs. ARIMA | -1.3849 | 0.2154 |
|  | LSTM vs. ARIMA | -1.5015 | 0.1839 |
